# The Response of Gray Treefrogs to Anesthesia by Tricaine Methanesulfonate (TMS or MS-222)

**DOI:** 10.1155/2013/635704

**Published:** 2013-10-01

**Authors:** Mary Paduano, Kaitlen C. Colafrancesco, Sarah A. Wong, Michael S. Caldwell, Marcos Gridi-Papp

**Affiliations:** 1 Department of Biological Sciences, University of the Pacific, 3601 Pacific Avenue, Stockton, CA 95211, USA; 2 Department of Ecology, Evolution and Behavior, University of Minnesota, St. Paul, MN 55108, USA

## Abstract

The design of anesthetic protocols for frogs is commonly hindered by lack of information. Results from fishes and rodents do not always apply to frogs, and the literature in anurans is concentrated on a few species. We report on the response of treefrogs (*Hyla chrysoscelis* and *H. versicolor*) to tricaine methanesulfonate. Body mass did not differ significantly between the species or between sexes. In the first exposure of a frog to TMS, variation in induction time was best explained by species (*H. chrysoscelis* resisted longer) and body mass (larger animals resisted longer). Multiple exposures revealed a strong effect of individual variation on induction time and a significant increase of induction time with number of previous anesthesia events within the same day. Recovery time was mostly explained by individual variation, but it increased with total time in anesthetic and decreased with induction time. It also increased with number of days since the last series of anesthesias and decreased with number of previous uses of the anesthetic bath. This is one of the first studies of anesthesia in hylids and also one of the first assessments of the factors that influence the variability of the response to anesthesia within a species.

## 1. Introduction

Anesthesia of frogs has been conducted mostly in scientific studies focused on physiology and more recently in taxonomic and ecological studies to allow for painless manipulation or euthanasia [1–5]. A variety of drugs and modes of administration have been used and comparative studies have revealed a great deal of variation in response among species [6, 7]. A well-informed choice of anesthetic and protocol leads to an anesthesia that does not harm the animal, maintains it sedated for the necessary amount of time, and is easy to handle [8].

Data on anesthesia in fishes and rodents can be applied to frogs only to a limited extent. Like fishes, frogs respond to anesthetics in a bath, but while fishes primarily absorb the drug through the gills, adult frogs lack such structures and absorb the drug through their permeable skin [9, 10]. In relation to mammals, amphibians metabolize and eliminate drugs at much slower rates because of their ectothermic metabolism [11]. Comparative studies are therefore necessary to assess the responses of amphibians across the range of available anesthetics and also across taxa. This will establish a basis for appropriate choices of anesthetics and protocols.

The anesthetics most commonly employed in frogs include benzocaine [12], tricaine methanesulfonate [7, 13], eugenol (clove oil) [14, 15], isoflurane [16, 17], propofol [18, 19], ketamine, and sodium pentobarbital [20]. Low temperature has also been claimed to have anesthetic effects on amphibians [6]. Several drugs have been tested specifically for analgesia, which targets the suppression of pain without affecting other sensations or motor control [6, 21–23]. These anesthetics and analgesics have been administered as injections, ointments, or most commonly as baths, taking advantage of the high permeability of the anuran skin [8, 23].

Tricaine methanesulfonate (TMS), also called MS-222, tricaine mesilate, or ethyl 3-aminobenzoate methanesulfonate, is among the most frequently used anesthetics in amphibians and fishes [8, 24, 25]. Its use has been greatly disseminated in the fish industry to reduce the metabolism of the animals during transport [9, 25]. Its main advantages are producing sedation with lower mortality than other drugs [7] and not requiring injection.

Studies have assessed the effect of TMS concentration on the response of amphibians, but have mostly focused on the genus *Lithobates* (=*Rana*) [10, 11, 20, 26–28] or on *Xenopus laevis* [4, 13, 29]. These animals belong to distantly related frog families (Ranidae and Pipidae) that diverged approximately 212 million years ago [30]. For reference, humans have diverged from mice only 92 million years ago. The long divergence time between these groups increases the likelihood that the differences in anesthetic response found between *Rana* and *Xenopus* exceed those seen between many anuran species. This is still reduced sampling within the clade of frogs, however, because it contains more than 5400 species divided in many families. Our study is focused on treefrogs (Hylidae), which form another distantly related group within amphibians, having diverged from the Ranidae 150 million years ago and from the Pipidae 212 million years ago. We report on the response of two sister species, *Hyla chrysoscelis* and *H. versicolor*, to anesthesia by TMS, and examine the effects of body mass, individual variation, sex, species, and repeated exposures.

## 2. Materials and Methods

### 2.1. Animals

Seven male and eight female Cope’s treefrogs, *Hyla chrysoscelis*, were captured in Carver county, Minnesota, and five male and six female gray treefrogs, *Hyla versicolor*, were captured in Wright county, Minnesota, during June and July of 2011. All animals were collected in amplexus with a conspecific of the opposite sex. Individuals of *H. chrysoscelis* were collected from a population that is allopatric with populations of *H. versicolor*. Pairs of *H. versicolor* were collected from a sympatric population and were positively identified as *H. versicolor* based on the relatively slower pulse rate of their trilled advertisement calls [31]. Collections followed Minnesota Department of Natural Resources permit number 17031 and IACUC protocol 0809A46721 to Mark Bee, University of Minnesota. The animals were transported together by airplane to the University of the Pacific, where they were housed individually or in pairs in 10-gallon terrariums. Each terrarium was supplied with misting and drainage of its peat moss bedding, a bathing pool which had its dechlorinated water replaced 3 times a week, a dark plastic hut for hiding, artificial green foliage, and a 3D structure made of PVC pipes for climbing. All terrarium contents were autoclaved or bleached before use. A diet of meal worms (*Tenebrio molitor*), crickets (*Acheta domesticus*), or large fruit flies (*Drosophila hydei*) was fed to the animals 3 times a week. In order to assess the potential effects of captivity, we included the time in the laboratory (75.3 ± 48.7 days) and the increase in body mass since arrival in California (1.5 ± 1.3 g) as predictor variables in our analyses but neither explained induction or recovery time significantly. The anesthesia procedures in this study gave support to measurements of auditory sensitivity (described below). All experiments were conducted at the University of the Pacific under M. G-P.’s IACUC protocols 10R08 and 10R09.

### 2.2. Anesthetic Bath

A bath was prepared at 23°C with TMS (Sigma-Aldrich) at 2 g/L in 100 mL of tap water aerated for at least 48 h for dechlorination and oxygenation. The solution was neutralized with NaOH, as TMS has been shown to be safer and more effective for amphibians at a neutral pH [27, 28, 32]. Each bath was used multiple times and we assessed the gradual weakening of the solution by including the number of previous usages of the bath as one of the predictors of induction or recovery time in the analyses described below.

### 2.3. Anesthesia

Each frog was placed in a transparent glass jar containing the 100 mL anesthetic bath. The animal could swim freely within the jar, without any restraining attachments. The degree of sedation was assessed through visual inspection of voluntary movements and through testing of the limb retraction reflex in response to gentle pinching of a toe with forceps at 1 min intervals. Induction time was defined as the time between immersion in the anesthetic bath and cessation of limb retraction in response to pinching. Once anesthesia was achieved, the subject was maintained in the anesthetic bath for an additional haphazard amount of time (7.6 ± 5.4 min) before being removed from the bath and momentarily rinsed with dechlorinated water to remove any TMS solution that could have remained on the skin. The purpose of this additional time was to extend the time to recovery. The total time in anesthetic is therefore the sum of the induction time and the extra time in the bath. The anesthetized animal was weighed, its snout-to-vent length (SVL) was determined with a caliper, and it was placed on a foam pad. Its skin was moistened every 5 min to prevent dehydration. The limb retraction reflex was monitored with regular gentle pinching of a limb every 5 min. Recovery time was defined as the time between removal from the anesthetic bath and the moment when the animal had recovered the limb retraction reflex and maintained the limbs symmetrically positioned in a normal posture.

The anesthesia events produced in this study were used to prevent movement of the subjects during noninvasive, painless measurements of hearing sensitivity. These included eardrum and body-wall vibration responses to sound and distortion-product otoacoustic emissions (sounds emitted by the ears). Instead of producing a single long anesthesia to obtain all the measurements, we opted for multiple events with shorter and lighter sedation. This reduced the risk of over anesthetizing the subject as the process could be easily interrupted, and it allowed us to assess the effects of multiple exposures in the animals’ response to TMS. The last anesthesia in each individual was used to prevent pain and movement during the surgical opening of a minute hole on the cranium. The hole was later used for neurophysiological measurements, which were followed by euthanasia.

### 2.4. Analysis

For the sake of simplicity, we employed a simple regression to describe the effect of body length on body mass and *t*-tests to evaluate the differences between sexes and species. The results were not qualitatively different from the results of an ANCOVA.

Our initial analysis of the response to the anesthetic was restricted to the first exposure of each individual to TMS, and we assessed the effects of body mass, sex, and species on induction and recovery time. An analysis of covariance (ANCOVA) was used to evaluate the effect of each fixed factor and body weight. Our second analysis included multiple exposures for each animal and we employed a general linear model to evaluate the effect of individual as a random factor nested within species or sex (fixed factors). As covariates, we examined the effects of body mass, number of previous exposures within the day, number of previous series of exposures in different days, number of days in captivity, change in body mass since arrival, and number of previous uses of the anesthetic bath. The percent variance exclusively explained by each independent variable was calculated as the sum of squares of the variable’s effect divided by the total sum of squares (effect + error). All analyses were conducted in IBM SPSS version 19. Mean ± standard deviation values are reported in the results.

## 3. Results

### 3.1. Body Size

**Figure 1:**
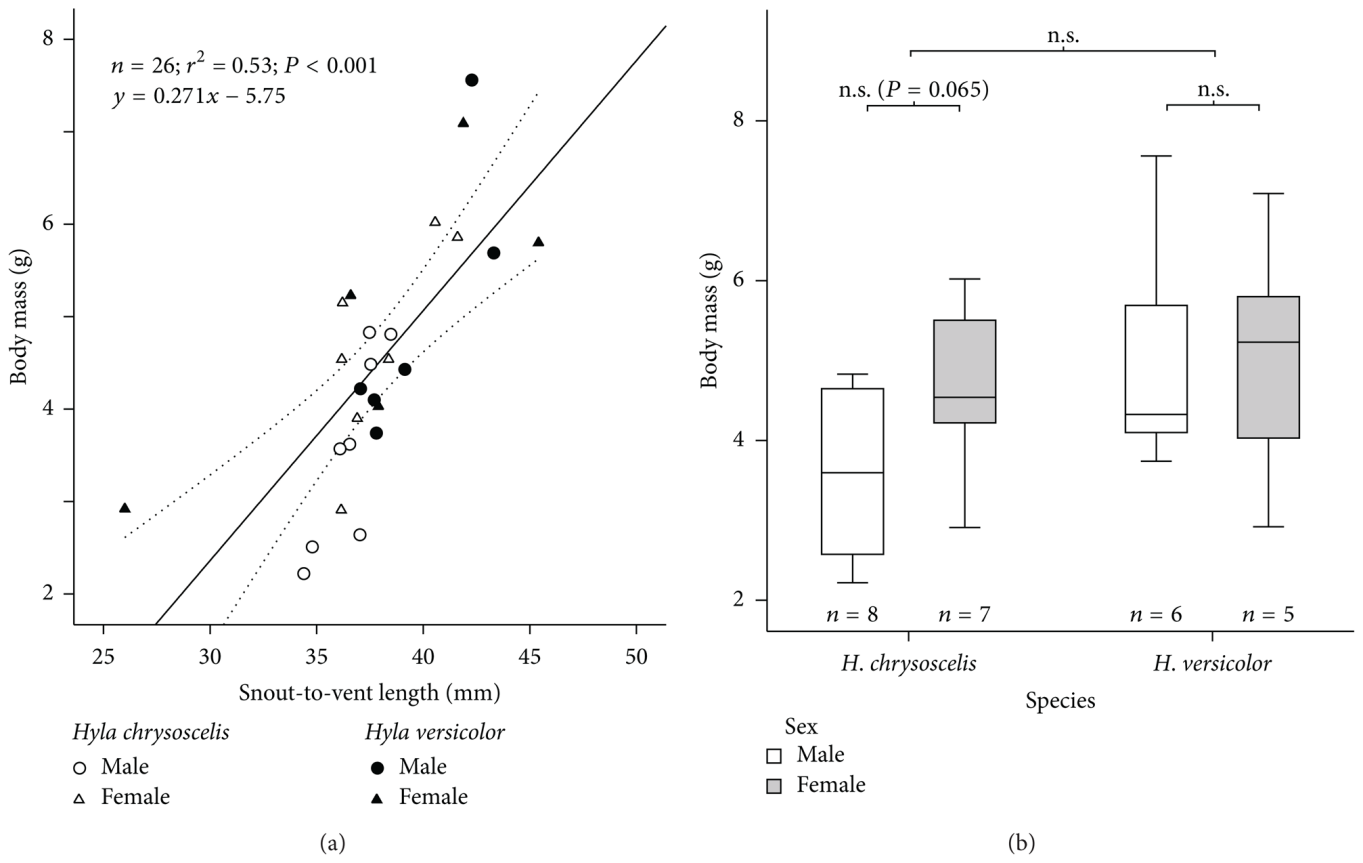
Body sizes of the treefrogs *Hyla chrysoscelis* and *H. versicolor* subjected to anesthesia in this study. (a) Relationship between body length and body mass. Dotted lines: 95% confidence interval of the mean. (b) Differences in body mass between species and sexes. n.s.: not significant.

Body length explained 53% of the variation in body mass of the gray treefrogs (Figure 1(a)). The difference in body mass between the two species was not significant, although *Hyla versicolor* (*n* = 11, body mass = 5.0 ± 1.4 g) was larger than *H. chrysoscelis* (*n*= 15, body mass = 4.1 ± 1.2 g; see Figure 1(b)). Within each species, body mass differences between the sexes were not significant either, although females in *H. chrysoscelis* (*n* = 7, body mass = 4.7 ± 1.1 g) were larger than males (*n* = 8, body mass = 3.6 ± 1.1 g).

### 3.2. Induction Time

**Figure 2:**
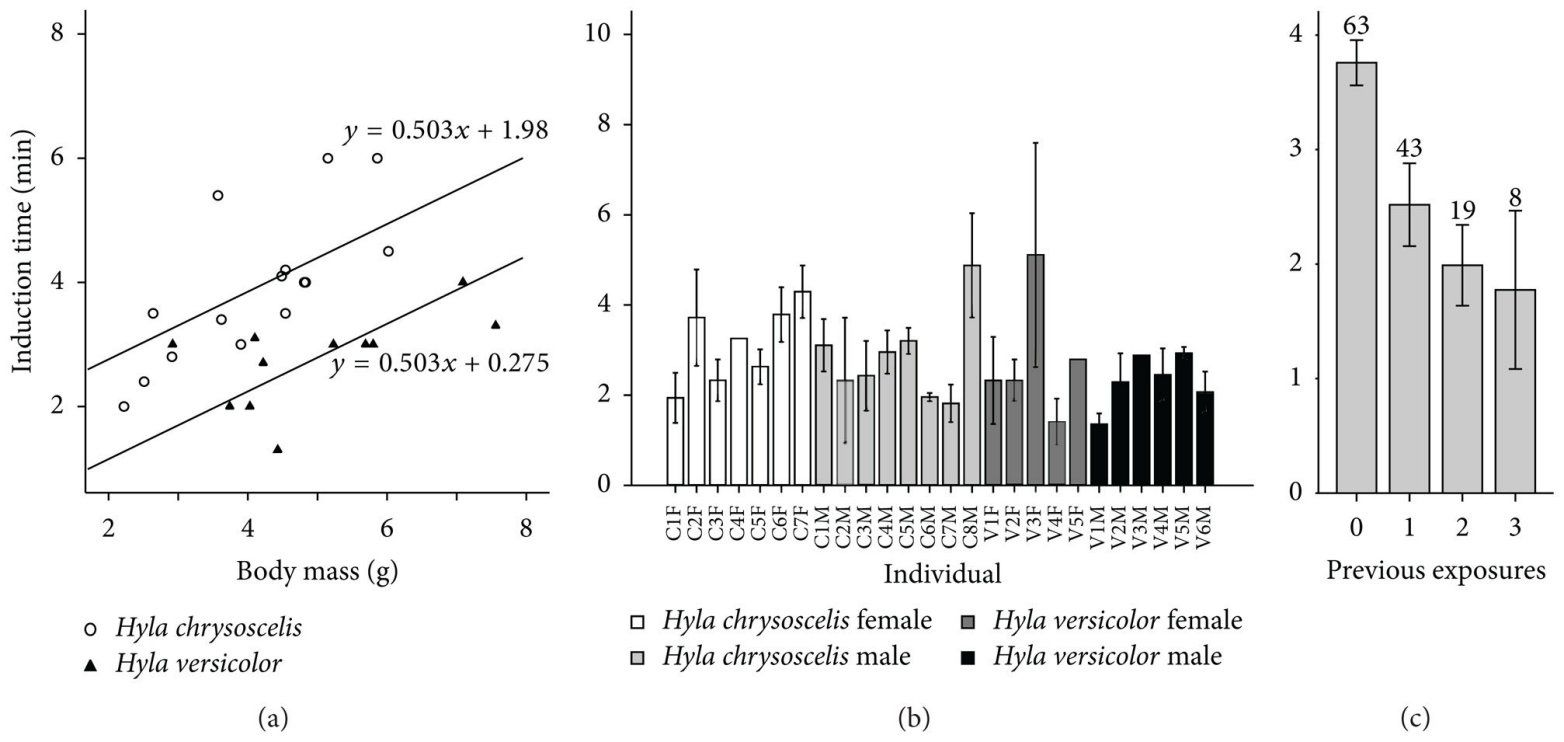
Induction time of gray treefrogs anesthetized with TMS at 2 g/L and pH 7. (a) First exposure of the animal to TMS. Both the slope of the relationship between body mass and induction time and the difference in intercept between the two species are highly significant (*P* = 0.001 and *P* < 0.001, resp.). (b) Individual variation in induction time across multiple exposures to TMS. (c)The effect of number of previous exposures to TMS within the same day on induction time. In (b) and (c), bars represent means and error bars represent standard errors. Numbers above bars represent count of anesthesia events.

In the first exposure to TMS, the induction time was 3.9 ± 1.2 min in *H. chrysoscelis* and 2.7 ± 0.7 min in *H. versicolor*. We assessed the effects of body mass, sex, and species on the response of the animals using an analysis of covariance. It revealed that species was the best predictor of induction time (*n* = 26, *F*_1,21_ = 20.8, *P* < 0.001; see Figure 2(a)). Independent of body mass, *H. chrysoscelis* resisted sedation by TMS 1.7 min longer than *H. versicolor*. The response of the gray treefrogs was also highly dependent on body mass (*F*_1,21_ = 13.3, *P* = 0.001) with each gram of increase in body mass adding 0.5 min to the induction time. Neither sex (*F*_1,21_ = 0.61, *P* = 0.6) nor its interaction with species (*F*_1,21_ = 0.18, *P* = 0.7) affected induction time significantly. Species and body mass, together, predicted 58% of the variation in induction time during the first exposure to TMS.

Multiple exposures to TMS allowed us to assess the effect of individual variation in addition to short-term (minutes to hours) and long-term (days) changes in the frog’s response to anesthesia. We employed an analysis of covariance with a nested design, to evaluate how well induction time is predicted by species and individual (nested within species). The covariates in the analysis included body mass, number of previous anesthesia events in the same day, number of previous days with anesthesia, number of previous uses of the TMS solution, and number of days since the subject’s arrival to the lab.

Individual variation in induction time was highly significant (*n* = 113, *F*_24,86_ = 2.7, *P* < 0.001) with the individuals’ mean induction times ranging from 1.45 min to 5.3 min (Figure 2(b)). The two species did not differ significantly in induction time (*F*_1,36.7_ = 0.37, *P* = 0.5). To assess the effect of sex, we repeated the analysis with individual nested within sex, instead of species, but we found that the difference in induction time between the sexes was not significant either (*F*_1,38_ = 2.8, *P* = 0.1). Repeated exposures did not have a significant effect across days (*F*_1,83_ = 0.01, *P* = 0.9), but within the same day, they were the best predictor of variability in the data set (*F*_1,86_ = 30.0, *P* < 0.001; see Figure 2(c)). Induction time decreased on average by 1.0 min with each anesthesia event, but the reduction was not linear, with the greatest difference occurring between the first and second events. The number of previous uses of the anesthetic bath (*F*_1,83_ = 0.78, *P* = 0.4) and the number of days since arrival of the subject to the lab (*F*_1,83_ = 0.52, *P* = 0.5) did not affect induction time significantly.

### 3.3. Recovery Time

**Figure 3:**
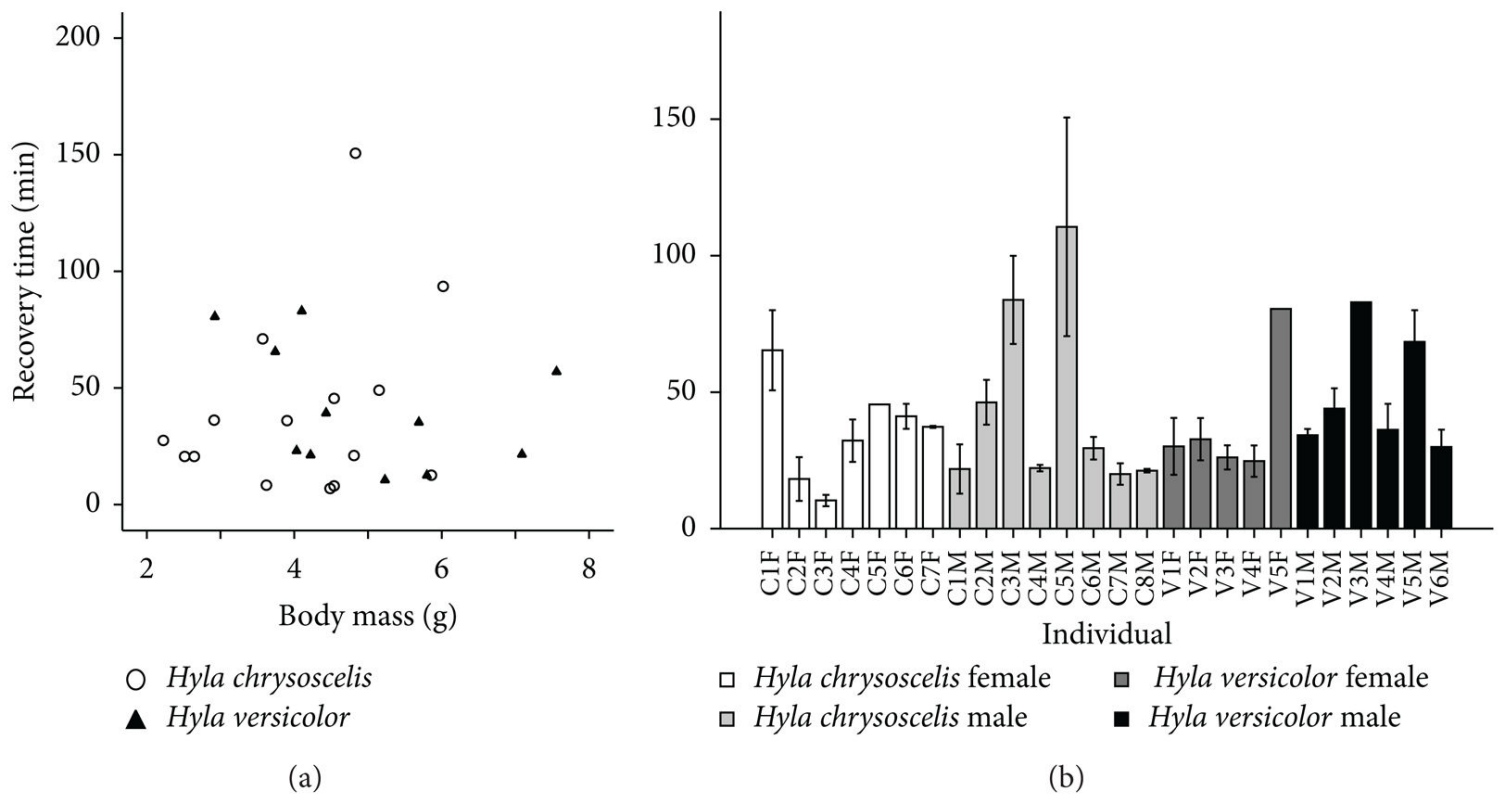
Recovery time of gray treefrogs anesthetized with TMS at 2 g/L and pH 7. (a) First exposure of the animal to TMS. Body mass and difference between the species did not have a significant effect on recovery time. (b) Individual variation in recovery time across multiple exposures to TMS.

**Figure 4:**
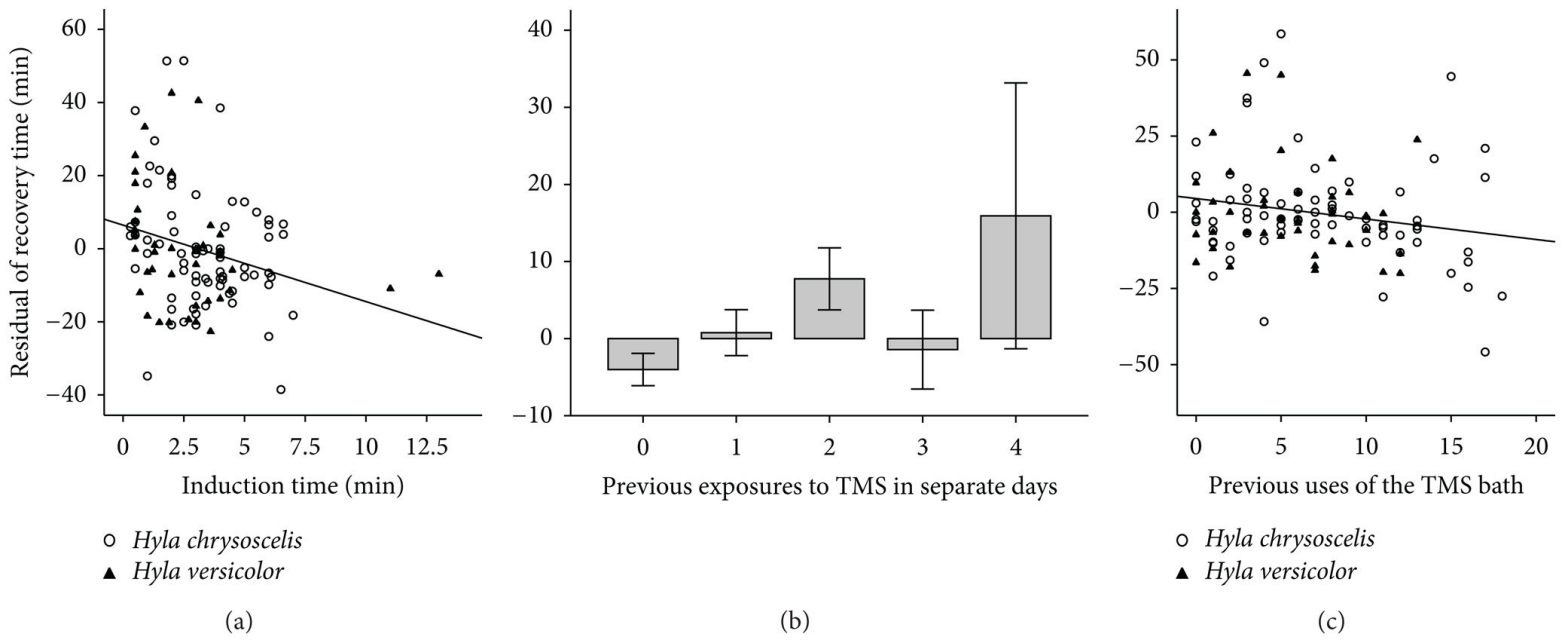
Factors influencing the recovery time of gray treefrogs anesthetized with TMS at 2 g/L and pH 7. (a) Induction time. (b) Number of previous days with exposure to anesthesia. (c) Number of previous anesthesias produced with the same solution of TMS. The ordinate of each plot is the residual of recovery time after the removal of the effects of all independent variables in the ANCOVA model except the one in the abscissa of the plot. Independent variables in the ANCOVA model included induction time, total time in the TMS bath, individual, species, previous days in which anesthesia was induced, and previous uses of the bath.

Following the first exposure to TMS, *H. chrysoscelis* recovered in 40.5 ± 39.0 min (*n* = 15) and *H. versicolor* recovered in 40.8 ± 26.5 min (*n* = 11). Recovery time was therefore more variable than induction time both for *H. chrysoscelis* (induction CV = 0.27, recovery CV = 0.96) and *H. versicolor* (induction CV = 0.29, recovery CV = 0.65) [28]. Body mass (*F*_1,20_ = 0.02, *P* = 0.9), sex (*F*_1,20_ = 1.0, *P* = 0.3), species (*F*_1,20_ = 0.1, *P* = 0.7), total time in bath (*F*_1,20_ = 1.2, *P* = 0.3), and induction time (*F*_1,20_ = 1.0, *P* = 0.3) did not affect recovery time significantly (Figure 3(a)). When multiple exposures were analyzed, individual differences formed the best predictor of variability in the dataset (*n* = 113, *F*_24,83_ = 6.4, *P* < 0.001; see Figure 3(b)). Recovery time was influenced by induction time (*F*_1,83_ = 11.1, *P* = 0.001; see Figure 4(a)) and by total time in TMS (*F*_1,83_ = 3.5, *P* = 0.067), although this effect did not reach significance, possibly because these two predictor variables were positively correlated with each other (*n* = 133, *r* = 0.24, *P* = 0.005). Recovery time increased with total time in TMS, but it had a negative relationship with induction time, indicating that animals that resisted anesthesia for a long time recovered from it more quickly. We examined the effects of species and sex separately by running the analysis (ANCOVA) with individual nested within species or sex. The effects of the other independent variables in the two analyses were almost identical, but neither species (*F*_1,28.8_ = 0.02, *P* = 0.9) nor sex (*F*_1,29.5_ = 0.9, *P* = 0.4) had a significant effect. Body weight (*F*_1,81_ = 0.0, *P* = 0.95) did not affect recovery time either.

The effects of repeated exposures to TMS on recovery time differed from the effects on induction time. Repeated exposures within the same day did not have a significant effect (*F*_1,81_ = 0.05, *P* = 0.8), but when examined across days, they significantly increased the recovery time (*F*_1,81_ = 7.9, *P* = 0.006; see Figure 4(b)). The number of previous days of anesthesia, however, also showed a positive correlation with the time since the arrival of the animals in the lab (*n* = 113, *r* = 0.60, *P* < 0.001) and with body mass (*r* = 0.24, *P* = 0.005). The animals were, therefore, gradually recovering less quickly from anesthesia and had gained weight in the lab. In addition, a bath of anesthesia was used repeatedly with the same animal, which made its number of uses also positively correlated with the subject’s time in the lab (*r* = 0.41, *P* < 0.001) and with the number of previous days with anesthesia (*r* = 0.56, *P* < 0.001). An ANCOVA evaluates the significance of an independent variable based on the amount of variance explained exclusively by this variable, so the measured effect of each of the two variables is reduced when they are correlated with each other. When all of these four variables were entered in the analysis simultaneously, the exclusive effects of number of previous anesthesia events (*F*_1,81_ = 4.9, *P* = 0.03) and number of previous uses of the bath (*F*_1,81_ = 10.9, *P* = 0.001; see Figure 4(c)) were significant, but those of time since arrival (*F*_1,81_ = 0.03, *P* = 0.9) and body mass (*F*_1,81_ = 0.0, *P* = 0.97) were not. When analyzed without the other correlated variables, time since arrival (*F*_1,84_ = 0.7, *P* = 0.4) and body mass (*F*_1,84_ = 0.0, *P* = 0.9) did not have a significant effect either. Recovery time was therefore increased by number of previous anesthesia events across different days, it was decreased as the number of uses of the anesthetic bath increased, and it was not affected by body mass or time since arrival in the lab.

The recovery time after anesthesia by TMS in this study could be predicted as

(1) recoverytime(min)=a+0.93b-3.50c-2.53d+8.22e+67.8,

where *a* is the effect of individual (from −63.8 to 54.0), *b* is time in bath (in min), *c* is induction time (in min), *d* is number of previous uses of TMS bath, and *e* is days since the last series of anesthesia events.

## 4. Discussion

Large individuals took longer than small ones to sedate in our study, but they did not take longer to recover. One would expect large animals to become sedated more slowly than small animals, because they have lower metabolism and because the skin area scales as a surface whereas body mass scales as a volume, leading to relatively smaller absorption areas at larger sizes [33]. In fishes, anesthesia by TMS tends to produce longer induction times and shorter recovery times in large animals than in small ones [34–36]. When the responses of the fishes are evaluated for other anesthetics in baths, however, this pattern is less clear, as body size has been found to exhibit direct, inverse, or no relationship with induction time [37–39]. In amphibians, body size was found to correlate positively with TMS induction time in the toad *Anaxyrus* (=*Bufo*) *fowleri* and the plethodontid salamander *Desmognathus fuscus*, but not in the hylid frog *Acris crepitans* or in the salamander *Ambystoma talpoideum* [7]. The authors of this comparative study pointed out that the lack of significance in *Acris crepitans* and *Ambystoma talpoideum* could be caused by their samples containing a narrower range of body masses than those for *A. fowleri* and *D. fuscus.* In another study, the effect of body size was assessed in African clawed frogs (*Xenopus laevis*) immersed in a bath of eugenol. Large individuals recovered more slowly than small ones, but their exposure time had been three times that of the small frogs, making it difficult to isolate the effect of body size [14].

Although the gray treefrogs in this study are sister taxa, they differ in number of chromosomes. *Hyla versicolor* is an allotetraploid, and the diploid *H. chrysoscelis* is one of its ancestral species [40, 41]. The two species have overlapping geographic distribution and body sizes and are very difficult to distinguish based on morphology [42]. Frogs and scientists in the field mostly rely on the pulse rate of the male calls, which is highly stereotypical and distinct, to discriminate between the two species [31]. Cell sizes are larger in the tetraploid [43], as a result of polyploidization, but the organism is not much larger, because the tissues are made of fewer cells [44–47]. Body size cannot account for the longer induction time that we observed in *H. chrysoscelis*, because *H. chrysoscelis* was slightly smaller than *H. versicolor*. It is tempting to hypothesize a causal link between polyploidization, cell size, and induction time in TMS, but additional data are necessary to rule out other unrelated physiological reasons for the difference.

Among hylids, the response to TMS has also been studied in cricket frogs (*Acris crepitans*) of 0.4–1.6 g body mass from North Carolina. The animals were immersed in unbuffered TMS solution at 0.5 g/L and showed an average induction time of 17 min and recovery time of 6 min [7]. This is a longer induction time than what we observed in gray treefrogs (3.3 min), which are larger animals (4.4 g), but we used a higher concentration of anesthetic (2 g/L) at neutral pH, which enhances the effect of TMS [28, 32]. Large species of frogs, in general, seem to exhibit longer induction times, but this relationship is obscured when distantly related species are compared. The available data for bufonid toads shows a gradual increase of induction time with body mass. Induction time was 38 min in the toad *A. fowleri* (2.1–27.3 g body mass) in unbuffered TMS at 0.5 g/L [7], 19.9 min in *Incilius* (=*Bufo*) *alvarius* (55.8–190.2 g body mass) in buffered TMS at 1 g/L [19], and 42.5 min in *Rhinella marina* (*Bufo marinus,* 130–250g bodymass) in buffered TMS at 3g/L [48]. Even heavier (450–750 g) ranid bullfrogs (*Lithobates catesbeianus* = *Rana catesbeiana*), however, took only 9.4 min to sedate in buffered TMS at 2 g/L [28].

In addition to body size and species, other biotic factors can potentially influence responses to TMS, but these have been understudied. Sex could influence the induction and recovery times in frogs [25]. Independent of any physiological differences between the sexes, indirect effects of sex can be expected through differences in body size or body composition, as egg masses can account for a substantial proportion of female body mass. In our study, however, sex did not affect the response to TMS significantly.

Studies with repeated exposures of amphibians or fishes to TMS are very rare, and the effects of individual variation in the response to TMS do not seem to have been assessed, although our results indicate that they are substantial. Data on the pharmacokinetics of TMS are crucial to understanding the potential short-term effects (minutes to hours) of repeated exposures. The Atlantic salmon (*Salmo salar*) exposed to a bath of TMS at 65 mg/L has shown a distribution volume of 3.98 indicating a rapid distribution of the drug to vascularized tissues [49]. The drug was eliminated rapidly with a half-life of 1.7 min and it could not be detected at 15 min. Plasma cortisol levels increased during induction, peaked during recovery at 30 min, and returned to normal levels within 360 min. This could be taken as evidence that the reduced induction times observed across repeated anesthesia events within a single day in our study were derived from stress as opposed to incomplete elimination of the drug from the body. In fishes, however, TMS is mostly absorbed and eliminated through the gills, whereas in frogs the skin fulfills an equivalent role [10]. In addition, we employed TMS at a higher concentration than Kiessling and colleagues [49], and this could have increased the physiological stress levels in the experiment. Data on TMS elimination measured directly in amphibians will, therefore, be necessary to identify the cause of the reduced induction times.

The gray treefrogs in our study recovered more slowly from anesthesia events that were preceded by anesthesia events on previous days. Their induction time was not affected by these previous anesthesia events though only by those produced within the same day. The only other study of the effect of TMS in multiple exposures focused on induction time and not on recovery time. Hybrid tilapias anesthetized in unbuffered TMS at 200 mg/L once a week for six weeks showed a gradual decrease in induction time [50]. The authors suggested that since TMS has been shown to be depleted from the tissues of many fishes very rapidly, the cumulative effect that they observed might be due to prolonged retention of some active products of TMS metabolism with unknown depletion time. This could also explain the increased recovery time in our study. A possible alternative is that the health condition of the frogs could have gradually deteriorated, but this seems unlikely, since they gained weight during the study and appeared healthy. Another possibility would be an increase in recovery time as the animal ages. Unfortunately, we could not obtain age estimates for our subjects to verify this idea.

While the response of gray treefrogs to anesthesia by TMS was more variable than observed in other species, the protocol was safe, producing no losses. Experiments in frogs and fishes comparing TMS to benzocaine and eugenol revealed that TMS allows for a substantially faster recovery of the subject and it produces fewer losses ([7, 20, 48, 51], but see [52]). A key point for these differences is that TMS is eliminated from plasma much faster than benzocaine or eugenol allowing the plasma levels of TMS to remain at lower levels and drop fast once the animal is removed from the anesthetic solution [10, 49].

Besides being administered in a bath, TMS has also been employed in anesthesia of amphibians as an intracelomic injection [26]. A major advantage of the bath in relation to the injection is that the treatment can be interrupted based on assessment of the subject. Overdose is not a concern, however, when TMS is used for euthanasia and TMS injections are more common in that context. While TMS is generally approved both for anesthesia and euthanasia, its use for euthanasia might require high concentrations and long exposure times for less sensitive species or use in conjunction with another drug, such as sodium pentobarbital [29, 53].

## 5. Conclusions

When treefrogs are anesthetized by TMS, (1) body size has a major effect on induction time but not on recovery time from anesthesia; (2) even sister species can exhibit differences in anesthetic response that are not explained by body size; (3) individual differences in response to anesthesia are substantial; (4) long induction times are associated with short recovery times; (5) repeated exposures produce a reduction in induction time when separated by minutes or hours and an increase in recovery time when separated by days.
